# Malaria eradication: the economic, financial and institutional challenge

**DOI:** 10.1186/1475-2875-7-S1-S11

**Published:** 2008-12-11

**Authors:** Anne Mills, Yoel Lubell, Kara Hanson

**Affiliations:** 1 Health Economics and Financing Programme, Health Policy Unit, Department of Public Health and Policy, London School of Hygiene and Tropical Medicine, Keppel Street, London, WC1E 7HT, UK

## Abstract

Malaria eradication raises many economic, financial and institutional challenges. This paper reviews these challenges, drawing on evidence from previous efforts to eradicate malaria, with a special focus on resource-poor settings; summarizes more recent evidence on the challenges, drawing on the literature on the difficulties of scaling-up malaria control and strengthening health systems more broadly; and explores the implications of these bodies of evidence for the current call for elimination and intensified control.

Economic analyses dating from the eradication era, and more recent analyses, suggest that, in general, the benefits of malaria control outweigh the costs, though few studies have looked at the relative returns to eradication versus long-term control. Estimates of financial costs are scanty and difficult to compare. In the 1960s, the consolidation phase appeared to cost less than $1 per capita and, in 1988, was estimated to be $2.31 per capita (both in 2006 prices). More recent estimates for high coverage of control measures suggest a per capita cost of several dollars.

Institutional challenges faced by malaria eradication included limits to the rule of law (a major problem where malaria was concentrated in border areas with movement of people associated with illegal activities), the existence and performance of local implementing structures, and political sustainability at national and global levels. Recent analyses of the constraints to scaling-up malaria control, together with the historical evidence, are used to discuss the economic, financial and institutional challenges that face the renewed call for eradication and intensified control.

The paper concludes by identifying a research agenda covering:

∘ issues of the allocative efficiency of malaria eradication, especially using macro-economic modelling to estimate the benefits and costs of malaria eradication and intensified control, and studies of the links between malaria control and economic development

∘ the costs and consequences of the various tools and mixes of tools employed in control and eradication

∘ issues concerning the extension of coverage of interventions and service delivery approaches, especially those that can reach the poorest

∘ research on the processes of formulating and implementing malaria control and eradication policies, at both international and national levels

∘ research on financing issues, at global and national levels.

## Background

The recent call for malaria elimination and, ultimately, malaria eradication raises many technical challenges [1]. However, the economic, financial and institutional challenges are at least as great. Economic challenges include analysing the level of investments appropriate for elimination programmes and in areas where intensified control is the only feasible option [2], and evaluating the relative return from investments in elimination and control, versus investments in other health interventions. Financial challenges include calculating the likely financial cost in terms of total cost and who pays, and putting in place funding mechanisms that can ensure the long-term support that will be vital if initial investments are not to be wasted. Institutional challenges are very diverse but involve the strength of the structures of state needed to ensure implementation, such as the rule of law (vital where malaria is concentrated in border areas with movement of people associated with illegal activities), the existence and performance of local implementing structures, and political sustainability at national and global levels.

The aims of this paper are threefold:

1. to review the evidence on the economic, financial and institutional challenges of previous efforts to eradicate malaria, with a special focus on resource-poor settings;

2. to summarize more recent evidence concerning these challenges, drawing on the literature about the difficulties of scaling-up malaria control and strengthening health systems more broadly;

3. to explore the implications of these bodies of evidence for the current call for elimination and intensified control.

A rapid literature review was undertaken of both historical and contemporary evidence on the economics and financing of eradication and control in resource-poor settings. Although what was termed eradication would now be called elimination, the word 'eradication' is retained here when reporting findings. Resource-poor settings are defined as those which historically or currently have limited physical and financial resources available to invest in malaria control, where the infrastructure of health services is limited, and where state capacity to implement health programmes is weak. Economic studies were identified from four previous reviews [3-6]. These were supplemented by a search in March 2008 for all relevant papers published up to that date, using four electronic databases (Pubmed; BIDs; ID21; HEED) and either MeSH terms where available (eradication, economic evaluation, cost/cost analysis, malaria) or text searches including the terms malaria and eradication, elimination, in combination with cost*; benefit*; effective*; econom*. References of identified papers were reviewed iteratively for further relevant studies. These searches related to economic and financial aspects were supplemented by historical literature on the institutional challenges faced by malaria eradication campaigns, and by contemporary evidence on constraints to scaling-up malaria control and health services more broadly, based on recent reviews [7,8].

Below the evidence is addressed in turn on the economics of eradication/control, on the financial costs, and on the institutional challenges. The paper concludes by presenting a research agenda.

## Economic analysis

Economic analysis of malaria control dates back as far as 1916, with a study documenting the economic impact of malaria on tenants on a Louisiana plantation [9]. For most of its early history, however, this type of studies can be characterized as anecdotal, often carried out to lobby the authorities to take action against the disease, or even to serve commercial or political interests, rather than to inform policy makers on efficient resource allocation [3].

When the 8^th ^World Health Assembly (WHA) launched the Global Malaria Eradication Programme (GMEP) in 1955, there was almost no documented evidence of the likely programme costs, nor analysis of the economic return from implementation. In 1958, the programme director wrote that eradication programmes were often initiated without adequate planning and cautioned against embarking on eradication attempts without full consideration of the necessary resources [10]. Dr. Pampana expressed his concern that spraying would be required over larger areas and for longer periods than initially estimated to break transmission, and that eradication programme costs would be higher than expected.

Since then, a variety of studies have been produced on the costs and consequences of what were called 'malaria eradication campaigns'. In selecting these for review, a distinction can be made between evaluations that aim to assess the overall value of a programme or strategy on economic grounds, referred to as *allocative efficiency*, and evaluations that consider the most *technically efficient *intervention for pursuing these. For the former aim, cost-benefit analysis (CBA) is the most appropriate tool, as it allows for direct comparisons, in monetary terms, of costs and benefits. This is especially important for a disease like malaria which has been shown to have not just detrimental health effects but also adverse consequences for economic development [11-13]. CBA can, at least in theory, encompass a broad range of both health and non-health related consequences including, for example, the benefits of improved educational capacity associated with reduced transmission, and the impact of this on productivity [12]. Technical efficiency can be examined through cost-effectiveness analysis (CEA), which can assess both the return in health terms to investments in different interventions, and the relative desirability of different approaches to delivering malaria prevention and treatment [14].

The focus of this review is on the former question, relating to the allocative efficiency of eradication campaigns and intensive control. Three methods are found in the literature for assessing their value. The most common approach is an assessment of a particular programme's costs and benefits; these are then summarized using a measure such as a benefit-cost ratio (BCR). Alternatively, the costs of an eradication campaign are compared to the alternative costs of long-term malaria control, with or without the inclusion of health outcomes. Lastly, assessments can be made at the level of the whole economy, either of the macro-economic impact of malaria control, or of the economic burden that malaria places on the entire economy. In relation to the latter, although this can then be compared to the costs of control or eradication, this comparison is often left implicit. Table 1 lists the studies, and where relevant their BCRs, in alphabetical order.

**Table 1:** Benefit/cost ratio (BCR) documented in the literature for eradication and control programmes.

**Source**	**Country**	**Focus of study**	**Design**	**BCR**
Barlow and Grobar (1986) [45] quoting Sudan (1975) [46]	Sudan	Control programme	No information	4.6

Cohn (1973) [19]	India	Anti-malarial programmes	Comparison of expenditure on control and eradication phases	NA

Griffith et al. (1971) [47]	Thailand	Chemopro-phylaxis	Comparison of benefits in terms of increased tungsten ore production and costs of chemoprophylaxis	6.5

Khan (1966) [48]	Pakistan	Cost of malaria	Estimated direct and indirect costs of malaria compared to cost of eradication programme	4.9

Livanadas and Athanassatos (1963) [49]	Greece	Eradication	Likely benefits due to morbidity and mortality avoided and, hence, improved agricultural output and economic performance and treatment costs avoided compared to eradication costs	17.3

Mills (1993) [16]	Nepal	Eradication	Retrospective analysis comparing costs of control with likely gains from economic development	Not calculated

Mills and Shillcutt (2004) [6]	Sub-Saharan Africa	Intensified control	Estimates of macro-economic benefits from (1) Gallup and Sachs and (2) McCarthy et al compared to control costs	4.7 (1) 1.9 (2)

Mills and Shillcutt (2004) [6]	Sub-Saharan Africa	Intensified control through package of interventions	Micro-economic analysis based on CEA evidence; costs compared to benefits estimated by valuing averted DALYs	17.1

Najera et al*(1993) [50]	Sri Lanka	Eradication	Macro-economic model of economy, tracing impact of control of malaria	146

Niazi (1969) [51]	Iraq	Eradication	Estimated direct and indirect costs of malaria compared to cost of eradication programme	6

Ortiz (1968) [52]	Paraguay	Eradication	Cost of eradication compared to estimated agricultural productivity gains	2.5–3.6

Ramaiah (1980) [53]	India	Control programme	Actual expenditure on control and eradication compared with likely economic benefits (reduction in direct and indirect costs)	2.4

Ruberu (1977) [20]	Sri Lanka	Intensive control aimed at eradication versus long-term control	Likely costs of eradication compared to direct and indirect costs of malaria	NA

*BCR calculated by [45] using data from [23]. NA: not applicable. Source: adapted and updated from [4].

### Cost-benefit analysis

In the years following the launching of the GMEP, a limited number of studies were carried out to evaluate national eradication and control programmes. Although the GMEP died out in 1969, along with the hopes of achieving global eradication, further evaluations were produced where the prospect of local elimination remained. Initially, the framework used by most of these evaluations was a CBA. In line with the dominant economic evaluation approach at the time [15], benefits were usually valued in terms of the human capital approach, and focused on benefits of malaria control in terms of increased labour supply and, hence, productivity and agricultural output (termed 'indirect benefits'), and reduced treatment costs (termed 'direct benefits').

The variety of settings and control measures, and the methodologies used in the evaluations, produced widely differing, though generally favourable, benefit-cost ratios, with the lowest being 1.9 and the highest 146 (Table 1). Moreover, since these analyses were primarily concerned with benefits of increased labour supply, they undoubtedly underestimated the benefits, which should also include the value of health gains *per se *and reduced suffering.

These studies were predominantly prospective, estimating likely costs of control and using information on the incidence of malaria and the workdays lost to estimate benefits. Even if studies used actual control costs, benefits were usually extrapolated based on known or estimated reductions in cases and deaths and an assumed relationship with increased agricultural output. In contrast, Mills [16] undertook a retrospective assessment of the relationship between the malaria eradication programme of Nepal and changes in economic indicators that might have been affected.

Historical evidence on Nepal sheds vivid light on the perceived dangers of malaria in the Terai area [17]. Malaria control started in 1955, and by 1968 only a few thousand cases were being detected annually. Control was subsequently maintained, though at a rather high level of cases. The research sought to track, from 1955 to 1985, both expenditure on malaria control and changes in the supply and productivity of labour, capital and land, as well as non health-related investments made in opening up new land for cultivation.

Table 2 summarizes the estimates of the gains and losses associated with malaria control, expressed per hectare of land; losses are relevant since many of the benefits were derived from migration, and allowance needs to be made for any losses in the source areas of migrants. The cost of malaria control (around Rs 25 per hectare per year) was insignificant compared to the returns to land and labour of new settlement (several thousand rupees per hectare), even taking into account non-health investment costs and losses in other areas. While it is impossible to rule out the possibility that pressure on land would have forced migration, even in the absence of malaria control, this would have been at a high cost in terms of morbidity and mortality, at least until deforestation and cultivation had made the environment less favourable for the main forest-dwelling vector and produced some measure of environmental control.

**Table 2:** The value of gains and losses associated with malaria control in Nepal (1980 prices).

**Category**	**Gains**	**Losses**
Marginal product of land	Max. of Rs 1,300 per ha and probably rather less	

Marginal product of settlers	Approx Rs 2,700 per ha	

Loss of marginal product of land in source areas of migrants		Unknown but probably small

Loss of marginal product of settlers in source areas		Approx Rs 1,200

Loss of marginal product of forested land		Minimum of Rs 220–400 per ha of agricultural land gained

Cost of malaria control		Average of Rs 25 per ha per year of control programme

Cost of agricultural investment		Rs 63–110 per ha per year

Ecological damage		Unknown

Gains and losses of indigenous population in settlement areas	Unknown	Unknown

Increased output and productivity of indigenous population	Small	

Costs associated with larger population		Only to a small extent attributable to malaria control

Nepali Rs 16.46 = £1 (1980). Source: [16]. Tab.2.7. from Chp.2 "The Impact of Malaria Control on the Economic Development of Nepal" by Anne Mills from "Health Economics Research in Developing Countries" By permission of Oxford University Press.

After the 1980s, cost-benefit analysis dropped out of fashion for a long time. The only recent CBA is that done by Mills and Shillcutt [6]. Following the recent approach of converting cost-effectiveness ratios to monetary values by applying a decision-maker's willingness to pay value to health gains, they drew on the cost-effectiveness literature to estimate the costs and averted DALYs of high coverage of a package of malaria control measures, and then calculated the BCRs by assuming a year of life gained is worth one per capita income. The resulting BCR for the intervention package was 17.1. This is likely to be an overestimate, since the health gains were derived from efficacy and effectiveness trials, which are likely to have more favourable results than in real life, and the costs probably allowed inadequately for system-strengthening costs. However, even given these caveats, the BCR is favourable.

Beside these cost benefit analyses, there is quite a large body of literature on the economic burden of malaria [12]. While using the human capital approach to valuing the costs of malaria, it differs from CBA by calculating losses due to malaria not the benefits of control, and by not including the costs of control in the analysis. A typical study of this type is that by Shepard *et al *[18], which summed total household and government expenditure on malaria treatment and productivity losses related to malaria morbidity and mortality based on costing from four African countries, and extrapolated to estimate the total burden of malaria in Africa, producing a total cost of 1.1 billion USD [18]. Chima, Goodman and Mills [12] critiqued the findings of this and similar analyses, demonstrating their shortcomings in adequately evaluating the true costs of malaria to the economy, notably the over-estimation of the productivity losses associated with uncomplicated malaria, and the underestimation of the economic burden of severe malaria[12]. Factors such as the impact of malaria on long-term educational attainment and subsequent productivity were also excluded.

### Cost comparisons

The second approach used to evaluate eradication campaigns is the comparison of their costs to those of ongoing control. Cohn [19] used this approach to evaluate the Indian eradication programme in the late 1950s. This approach was particularly relevant given the argument put forward by WHO and adopted by the Indian government, that a high investment in the short run on an eradication campaign would be more economical than long-term annual investment in control. By comparing the expected costs of a 10-year eradication campaign to an annual control programme run for 30 years, Cohn demonstrated that the cost of eradication can be higher than that of control, depending on the discount rate used for adjusting future expenditure to its present value. In the Indian context, at a discount rate above approximately 10%, control appeared to be the more economical option.

Health outcomes were not included in the analysis, though Cohn did discuss the consequences of eradication in terms of reduced morbidity and increased fertility leading to rapid population growth, particularly in the younger, economically-dependent age groups, and its possible detrimental effects on the economy. He also argued that the proposed reduction in morbidity and the contribution of this to the economy was overstated, due to unsound methodologies, and that most of the potential gains had already been reaped through routine control.

In contrast, Ruberu's estimates for Sri Lanka suggested that the costs of a high short-term investment on eradication would be exceeded both by the costs of long-term control, and by the productivity losses if no action were to be taken [20]. Ruberu allowed for the need to switch to higher-cost insecticides given the likely development of resistance with prolonged spraying programmes.

Most recently, Jackson, Sleigh and Liu [21] described the costs associated with the malaria control programme in Henan, China, where reported malaria cases had dropped from over 10 million in 1970 to 318 in 1992. Henan achieved the consolidation or 'basic elimination' phase in the following year. Much of this reduction has been attributed to the use of insecticide-treated nets (ITNs), which almost eliminated the primary vector, *Anopheles anthropophagus *[22]. The support to the use of ITNs, however, was discontinued in the early 1990s due to financial constraints, and since then a number of outbreaks had occurred with the number of annual cases ranging in the thousands.

The Henan study provides estimates for both government expenditure on malaria control – approximately $0.03 per person protected – and for the income losses and treatment costs for patients with suspected malaria – just over $4 (in 2002 USD). The authors did not estimate a BCR or evaluate the case for further investment to pursue full elimination. They did however conclude that discontinuing the malaria control programme would likely result in considerably higher costs.

### Macro-economic analysis

The third approach to assessing the value of malaria eradication is by evaluating the impact of malaria on the economy as a whole. This methodological approach can be justified as the most appropriate of all three, given the pervasive effects of malaria on an economy [13]. The first such evaluation was Barlow's analysis of the economic impact of near-eradication of malaria in Sri Lanka [23]. His macroeconomic model produced a positive impact on output in the first decade, but, in the long term, the growth of output would be outstripped by the growth of the population, reducing income per capita. However, the demographic consequences of both malaria and malaria eradication has been an issue of considerable controversy in Sri Lanka [24,25].

Two more recent studies have assessed the overall burden malaria places on the economy by assessing its impact on rates of economic growth. Gallup and Sachs [26] used malaria as an explanatory variable in an economic growth model. Their results suggested that countries with 'intensive' malaria grew 1.3% less per person per year, and a reduction of 10% in malaria was associated with 0.3% higher growth. McCarthy, Wolf and Wu [27] did similar estimations, but allowed for two-way causality between morbidity and economic growth rates. This resulted in a lower estimate: a 0.25% per year reduction in economic growth and far greater variability in results across countries.

Neither study matched these economic growth estimates with control costs. However Mills and Shillcutt [6] applied the relationship between malaria and economic growth to estimate the increased annual economic growth rate associated with a 50% reduction in the malaria burden (the Abuja target), and then calculated the BCRs by comparing the gain in national income to the costs of high levels of coverage of a package of malaria control measures. BCRs of 4.7 and 1.9 suggested that malaria control was a good investment.

## Financial costs

The historical evidence on the financial costs of eradication and intensified control is rather thinner than the economic analysis literature reviewed above. It is also difficult to summarize, given that costs depend greatly on the stage of the programme – whether the costs relate to the initial attack phase or to the consolidation phase. However, some idea can be obtained by using the cost data from economic analyses of the large-scale programmes in Thailand and Nepal, and country data put together by Griffith [28] when the GMEP was moving into the consolidation phase in a number of countries (Table 3). The difference between the Griffith data (range in 2006 USD: 0.45–0.84) and that for Thailand and Nepal may reflect the fact that for the former data, consolidation came just after the successful reduction of cases during the attack phase, whereas the latter data come from the 1980s when the eradication goal had been abandoned and countries were struggling with persisting malaria in specific geographical areas. In 1988, the annual cost of intensive, comprehensive control was estimated at the 2006 USD equivalent of 2.31 per capita [29].

**Table 3:** Historical evidence of the financial costs of the consolidation phase* of malaria elimination.

**Country**	**Annual cost per person protected (in 2006 USD)**	**Source**
Taiwan	0.45	Griffith 1961 [28]

India	0.50	Griffith 1961 [28]

Sri Lanka	0.74	Griffith 1961 [28]

Indonesia	0.84	Griffith 1961 [28]

Thailand	1.33	Kaewsonthi and Harding 1984 [54]

Nepal	0.64–1.03**	Mills 1992 [55]

Source: adapted from Table 4 in [4]; 1984 values updated to 2006 using the US GDP deflator. *Involving surveillance, treatment of cases, and spraying when needed; **range represents areas of different endemicity.

On a per capita basis, these costs look surprisingly modest. However at the time malaria eradication was being attempted, expenditures absorbed a sizable share of government health expenditure. In India, for instance, Cohn calculated that the expenditure on eradication during the campaign was one third of total health expenditure, and in Nepal, malaria control in the 1960s and 1970s accounted for 20–25% of Ministry of Health recurrent expenditure [3].

Little specific information is available on sources of funding. In Nepal, Mills [3] found that between 1955 and 1985, external sources funded insecticides, drugs, equipment and vehicles, while the government financed local costs, primarily salaries. In general, around 56% of costs were locally financed, though this share depended greatly on how much insecticide was provided, since this was quite costly. Evidence in Griffith [28] suggests that this picture on the balance of internal and external funding was mirrored elsewhere.

It is commonly said that the need to fund surveillance adequately was underestimated by the GMEP, and that the less visible malaria became as a problem, the more difficult it was to maintain expenditure. Indeed, the Eighth Report of the Expert Committee on Malaria [30] suggested that experience indicated that 'a well-operated consolidation mechanism costs, per annum, 65% to 75% of an attack mechanism'. Griffith noted that 'to the eradicationist, the need to maintain financial support at a high level, when malaria has been reduced to a low level, must often be seen the supreme fiscal headache surmounting the large body of administrative and technical problems to be overcome for a successful termination of the eradication programme', a point highly pertinent to current discussions about elimination.

Recent analyses of the costs of malaria control began with the work of the Commission on Macroeconomics and Health (CMH). The CMH estimated the costs of achieving 70% coverage of interventions for the prevention and treatment of malaria in all countries with GNP per capita of less than $1,200 in 1999 USD (a total of 83 countries, not all of which were malaria affected). Total annual costs (in 2002 USD) of prevention and treatment of adults were $3,535 m – 5,267 m, or $0.74–1.1 per capita of the total population [31], plus a share of the $9,414–11,987 m ($1.97–2.50 per capita) cost of treatment of childhood diseases including malaria. These costs included the direct capital and recurrent costs of the interventions plus the support and supervision needed at a district level. Allowing for the costs of broader system strengthening needed at higher management levels to support malaria control might double these costs [32]. Kiszewski *et al *[33] similarly estimated the total costs of scaling-up a set of malaria control measures, but in the 81 countries most heavily affected by falciparum malaria. They included both service and programme strengthening costs, and 100% coverage targets. Total annual costs of fully scaled up services were $4,468 – 5,660 m (in 2006 USD), or $2.35–2.98 per capita of populations in falciparum-affected areas.

The most recent estimates are from the Bill and Melinda Gates Foundation's commissioned projection of resource needs for the global malaria effort, and the Roll Back Malaria (RBM) Global Action Plan published in September 2008. The estimate presented at the Malaria Forum in Seattle was $6 bn annually for implementation costs of 80% coverage in 107 countries covering 3.2 bn people at risk of falciparum and vivax malaria ($1.88 pc). RBM's Global Malaria Action Plan estimated the costs of country implementation of malaria control and elimination strategies to be $5.3 and 6.2 billion ($2008) in 2009 and 2010, respectively, and $5.1 bn per year from 2011 to 2020, for 109 countries and 3.3 bn people at risk, suggesting roughly $1.55 per person [34].

Cost comparisons are fraught with problems. In particular, the above estimates differ in terms of countries included, target coverages and costing approaches, and costs in any specific country setting will vary greatly depending on the appropriate mix of interventions. Nonetheless the studies suggest that around US$1.5–3.0 per capita per year are required to maintain malaria control efforts. The RBM estimates, which project costs well into the future, do not anticipate costs falling as a reflection of elimination successes until after 2020.

## Institutional challenges

Malaria eradication campaigns in Asia generally began when coverage of routine health services was limited. For example, in Nepal, malaria eradication was the first health programme to reach into villages, and efforts were not made to strengthen basic health services until the early 1980s. The vertical nature of malaria eradication programmes – involving spraying teams and active case detection and treatment, managed by a self-contained organization – meant that programmes created and maintained were largely independent of the rest of the health service infrastructure.

Nonetheless, spraying programmes struggled to operate efficiently. Common problems in countries such as India, Nepal and Sri Lanka included: delays in starting annual spraying campaigns, so that spraying occurred after the rise in numbers of cases at the start of the transmission season [3]; high levels of refusals by house owners to have their houses sprayed and replastering of walls following spraying [35,36]; and shortages of insecticide resulting in sub-optimal levels of coverage [3], which were aggravated by increasing reliance on the more expensive and short-acting insecticides as a result of the development of resistance to DDT. More generally, insecurity, war and armed political struggles were major obstacles in large areas of countries [37].

From the 1970s, there was declining external support for malaria control as enthusiasm waned amongst donors for providing continuing assistance. With a change in policies favouring primary health care and decentralization, there was increasing pressure to integrate malaria control with other health programmes to capture efficiencies and synergies. Thus, in Nepal, malaria control was, over time, integrated with the community health development programme, and community health workers were expected to undertake a number of tasks. Experience with integration in a number of countries suggests that the efficiency of malaria control suffered with integration, and also that multi-purpose workers were poorly trained and supported to take on a broad range of tasks [37]. Another common problem was that decentralized services lacked technical expertise in areas such as malaria and control of other diseases [38].

A further institutional problem was that in some countries with relatively good control programmes, cross-border traffic maintained the malaria problem. Although the Nepalese control programme was not fully effective, it was more effective than in adjacent Indian districts, and imported malaria remained a constant problem. In Thailand, where control was more efficient, nonetheless border malaria remained a major problem because of the circumstances of Cambodia and Myanmar, with flows of people affected both by conflict and by opportunities for profitable, but illegal, productive and trading activities, such as forestry and gem mining. Part of the problem was also a continuation of malaria in remote and difficult to reach areas with poor roads. The far-west of Nepal, for example, was never fully included in the eradication effort due to problems of physical access.

Recent efforts to scale up malaria control interventions have taken place in a more complex environment of pluralist health-care delivery systems and ever-expanding donor initiatives, such as the Roll-Back Malaria Partnership, the Global Fund to fight AIDS, TB and Malaria (GFATM), the World Bank Malaria Booster Programme, and the US President's Malaria Initiative (PMI). Whilst bringing dramatically expanded resources for malaria control, this proliferation of global initiatives has placed new demands on programme managers in terms of coordination, reporting and accountability requirements.

The challenges currently facing malaria control are in many ways indistinguishable from those facing current health systems as a whole. Drawing on a framework developed for the Commission on Macroeconomics and Health to classify the health system constraints to scaling-up priority health interventions [7,8], Table 4 sets out the levels at which these constraints operate and gives examples of the problems in relation to scaling-up malaria control interventions. Malaria-related constraints can be identified at all levels, from household and community-level constraints on demand for effective interventions, through to civil service rules and other cross-sector government policies that restrict efforts to improve the remuneration and performance management of health workers.

**Table 4:** Current challenges to scaling-up malaria control.

I. Community and household	Limited demand for malaria prevention and treatment due to lack of information, high cost, or physical inaccessibility; lack of community engagement in malaria control.
II. Health service delivery	Shortage and maldistribution of appropriately qualified staff; inadequate supply of consumables including malaria drugs, diagnostic tests, insecticide; lack of equipment and infrastructure including poor accessibility of health services; poor-quality diagnosis and treatment in public and private sectors; weak technical guidance, programme management and supervision; inequities in programme reach.

III. Health sector policy and strategic management	Weak national malaria control programmes; weak drug policies and supply systems; inadequate communication with the private sector and regulation of retail drug sales and fake drugs; weak incentives to use inputs efficiently and respond to user needs and preferences; reliance on external funding reduces flexibility and ownership; donor practices overload country management capacity

IV. Public policies cutting across sectors	Taxes and tariffs on import of malaria-related commodities; decentralization policies place responsibility where technical capacity is weak; inflexible government bureaucracy (civil service rules and remuneration; centralized management); poor availability of communication and transport infrastructure

V. Environmental and contextual characteristics	Governance and overall policy framework: corruption, weak government, weak rule of law and enforceability of contracts; political instability and insecurity; low priority attached to social sectors; weak structures for public accountability; lack of free press. Physical environment: climatic and geographic predisposition to disease; physical environment unfavourable to service delivery.

Adapted from [7].

Of particular relevance in the current climate is the issue of whether intensified malaria control initiatives should be vertically organized, working within malaria-specific management and supply structures, or integrated with the broader health system. The vertical approach has the advantage of providing focused technical input and resources and, potentially, quicker progress in the specific disease area. However, a variety of experiences have demonstrated how such approaches can lead to duplication of effort, distortions among programmes, and disruptions in the delivery of routine health services [8]. Travis *et al *have argued that a health systems approach to addressing the root problems which are shared by all disease control programmes is potentially more efficient [39].

The horizontal/vertical discourse has recently evolved towards advocacy for a "diagonal" approach [40] in which disease-specific priorities and resources are used to drive through health system improvements which tackle shared problems such as human resources, health financing, drug supply and information systems. However, there is so far limited evidence of the success of such approaches.

## Implications for malaria elimination and intensified control

What can be drawn from the bodies of evidence reviewed above with respect to the economic, financial and institutional challenges that face the renewed call for elimination and intensified control?

### The economic return from malaria elimination/control

As is clear above, the bulk of the cost benefit analyses date from the eradication era, and suggest that the benefits of malaria elimination and intensified control exceed costs. However, there are a number of caveats with respect to the relevance of these findings for the present day. Firstly, BCRs for elimination remain largely hypothetical – they are mostly prospective, and have not been followed up later to assess how close to the truth they came. It is highly likely they were optimistic with respect to both the level of costs and the period over which continued control would be necessary. The retrospective assessment by Mills of malaria control in Nepal [14-16] is the only study that sought to explore, within a country setting, what benefits might reasonably associated with malaria control. In addition, the Gallup and Sachs and McCarthy *et al *studies demonstrate that countries with less malaria historically had higher rates of economic growth, but there are questions on how appropriate it is to extrapolate this conclusion to the control of malaria in the high-burden countries of sub-Saharan Africa and South Asia.

Secondly, the early literature is preoccupied with the implications of elimination for population growth. Again, there appears to have been no retrospective assessment of whether the fears were well grounded or not, but there is now greater understanding of the relationship between improved health and population demographic change, such that demographic implications are not now employed as a reason to ignore disease burden.

Thirdly, the underlying cost structures of malaria control are likely to be somewhat different. It might be expected that the relative prices of physical and human inputs to malaria control are now very different from those during the era of eradication, though this remains a subject for further investigation. The range of interventions is now broader, encompassing long-lasting insecticidal mosquito nets as well as intermittent treatment options for pregnant women, infants and children, and the delivery modes are more varied. Social marketing approaches for example, and piggy-backing interventions on the retail sector, are not delivery modes that were previously available. The much greater availability and use of private sources of treatment increases household costs, but might be expected to reduce the impact of malaria elimination and control on health. As Cohn argued, it is possible that, in some settings, most of the health gains associated with elimination may have been, or can be, captured through ongoing control. This would imply that the key comparison is financial – the net costs of control versus the net costs of elimination.

Finally, in many areas, malaria was associated with certain environmental features, which have changed with increased population growth: deforestation, expanded cultivation and urbanization. In addition, increased incomes and related socio-economic development have improved housing and sleeping conditions, knowledge of personal protection measures, and ability to purchase health care. Thus the potential for malaria transmission, and also for malaria reduction, is now less in many areas. This changes both costs and benefits.

### Financial challenges

Although the cost estimates presented above are approximate, it seems that expenditure of the order of a few dollars per capita is likely to be required for intensified control measures, though additional funding would be required to finance the high-level systems support necessary for interventions to be delivered efficiently and at scale. Given the large number of people at risk of malaria, such per capita sums translate into very substantial total financial requirements.

A much greater challenge, however, is likely to be maintaining donor and national government support for the period of time required for elimination, or merely to maintain high coverage of control measures over time. The history of immunization demonstrates how international efforts have waxed and waned over time, and the history of malaria eradication itself demonstrates its vulnerability to decreasing interest over time. Funding mechanisms are needed that protect countries from short-term fluctuations.

### Institutional challenges

In environments such as the bulk of sub-Saharan Africa, where expanded coverage of existing control measures is the immediate target, the main challenges are those outlined earlier: overcoming the constraints at household, health-service delivery and policy levels, and at cross-sectoral level, including issues of governance and accountability.

The evidence on the costs and effects of strengthening intervention delivery and coverage through a health systems approach remains limited, though some promising approaches have been identified [41], which also have the potential to strengthen malaria control activities. For example, improved performance at local level has been obtained by strengthening structures of accountability to communities and introducing mechanisms to ensure that users have a voice in the local health system and can influence priorities. In Burkina Faso, participation by community representatives in public primary health care clinics increased coverage of key interventions. In the area of human resources, there have been some successful experiences of using mixes of financial and non-financial incentives to improve provider performance. In terms of organization of service delivery, there are also some positive examples. Contracting NGOs to deliver primary care services in Cambodia is one example.

Of particular relevance to malaria control is the increased pluralism and complexity of contemporary health systems compared to those existing in the eradication era, particularly with respect to the volume of private providers of all types. Governments cannot now ignore the role that private providers play in malaria treatment, for example, and need to identify ways to work with private providers to improve the quality of services they provide. Although a number of promising approaches have been identified, the evidence remains patchy [42]. The contemporary international environment is also much more complex than in the days of the GMEP – there are many more players, with the possibility for conflicting guidance and certainly competition between various disease control initiatives for scarce resources, such as trained health workers.

In those parts of the world where elimination of malaria is technically feasible in the short- to medium-term, epidemiological and technical differences will influence the institutional responses needed at different levels of the system (Table 5). First, changing epidemiology will affect people's perception of the severity of illness and the acceptability of control measures. For instance, as the perceived risk of malaria diminishes, support for vector control measures, such as spraying, may wane. Effective communication and community engagement will be needed to ensure continued high coverage of interventions. In addition, while the current focus on reaching the poor with interventions is driven by equity concerns, elimination will bring an additional epidemiological impetus to providing services in these communities and possibly fuel efforts to reach isolated communities more effectively.

**Table 5:** Additional challenges for elimination and intensified control.

I. Community and household	Reduced acceptability of control measures such as house spraying as perceived malaria risk falls; acceptability of new interventions such as mass screening and treatment; changed behavioural response to illness as age distribution of malaria changes and malaria share of fevers declines; access barriers to reaching isolated (geographically, socially, etc.) communities for outbreak control
II. Health service delivery	Need for effective disease surveillance and response systems

III. Health sector policy and strategic management	Strengthened links between technical programmes (e.g. malaria and MCH programmes) to ensure revised guidance for appropriate management of non-malaria fever and greater burden of disease in adults

IV. Public policies cutting across sectors	Legal frameworks and border controls for coordinating action in cross-border outbreaks; financing mechanisms that allow for and protect commitments to malaria control

V. Environmental and contextual characteristics	Ensuring sustained political and financial commitment to eradication at all levels; structures of public accountability that will support such commitment

Second, emphasis at the service-delivery level is likely to shift from passive detection and case management to more active screening and treatment strategies, requiring strong surveillance systems. This will be facilitated by modern communications, which will make it easier to transmit information about disease outbreaks and elicit appropriate control responses. Means of including private providers in these systems will need to be identified.

Third, at the health policy and management level, structures within health ministries will need to adapt to allow for more effective communication and coordination between malaria control programmes and other technical programmes, in order to ensure that technical guidelines, such as those for IMCI, are adapted to a shifting disease burden. The growing body of evidence about diverse ways of organizing service delivery, and greater awareness of the need to combine the technical expertise of disease control programmes with an integrated approach to service delivery, should help to support effective and sustained control programmes.

At the fourth level, intersectoral collaboration will be needed to develop appropriate mechanisms to manage cross-border outbreaks. New financial mechanisms will be needed to ensure that adequate resources for elimination are allocated, and that these allocations to malaria control are protected as the perceived risk diminishes. Sustaining political and popular support for malaria eradication is likely to be a major challenge.

## A research agenda

Research in recent years has concentrated on developing the evidence-base on the cost-effectiveness of malaria control interventions, in order to demonstrate that malaria control represents value for money, and on evaluating approaches for scaling-up these interventions. The goal of eradication, however, changes the context and poses somewhat different needs for evidence.

Firstly, the question of the allocative efficiency of malaria eradication assumes centre stage. If substantial resources are to be devoted to malaria over the long term, then the case for these investments can be greatly strengthened if it can be shown that benefits will substantially exceed costs. Recent work has made a persuasive case that the effects of malaria are such that a macro-economic framework is needed to assess costs and benefits. The value of this approach is also supported by recent work on the macro-economic consequences of antimicrobial resistance [43]. A key priority is to develop a dynamic macro-economic model which can explore the economic consequences of malaria eradication and differing levels of control, taking into account recent evidence on the broader economic consequences of malaria, including its impact on the intellectual development of children [44].

Such modelling, however, needs to be underpinned by a greater volume of research on the connections between malaria control and economic development. Evidence is still very limited on many of the potential benefits of reduced malaria, such as human capital improvement, labour supply, agricultural production, foreign investment and tourism.

Secondly, there are large information needs related to the costs and consequences of control and eradication. Virtually none of the existing literature on the cost-effectiveness of interventions takes into account the impact of scale on cost-effectiveness, or the cost-effectiveness of combinations of interventions. Priorities for research include:

∘ the cost-effectiveness of interventions as the level of transmission changes over time (employing dynamic cost-effectiveness modelling)

∘ the cost-effectiveness of packages of interventions, including evidence about the extent of economies of scope and/or synergies in effectiveness

∘ the cost-effectiveness of "new" strategies such as active case detection and mass screening and treatment after the intensified control phase is over, including modelling to determine the optimal transmission levels at which to change strategy

∘ the incremental cost-effectiveness of proceeding to elimination once a high level of control has been achieved.

Thirdly, there is a substantial research agenda around extending coverage of interventions and issues of service delivery. Eradication will require malaria control programmes to reach into all parts of a country. Research is needed to identify new ways to extend the reach of malaria control interventions, to enable programmes to extend their coverage of marginalized and physically isolated populations. This should include examination of the costs and effectiveness of multipurpose programmes compared with malaria-specific ones, and the appropriate combination of disease-specific and integrated service-delivery models to allow both effective disease control and to maximize the positive impacts on the health system as a whole.

Fourthly, research is needed on the processes involved in formulating and implementing malaria control and eradication policies, at both international and national levels. Gramiccia and Beales [37] commented that 'one of the important shortcomings of the eradication programme was the incapacity or unwillingness of some governments to support and manage their own national programme. This applies particularly to those governments that had been pushed into eradication by international pressure or incentives'. Policy analysis research at national and global levels can help to understand the political landscape, and identify how political strategies might be crafted to enable longer-term political support in both donor countries and endemic countries. Research on the processes of policy implementation can help to understand why agreed policies can fail to be implemented at the local level, and help design improved implementation processes.

Finally, research is needed on the financing issues, including approaches to protecting long-term financing commitments, ways to ring-fence resources (at national and global level) over time and methods for estimating resource requirements and how they might change over time.

## Competing interests

The authors declare that they have no competing interests.

## Authors' contributions

AM drafted the overall paper; YL reviewed the historical evidence and contributed relevant sections; KH drafted sections on current constraints, future challenges, and research priorities. All authors reviewed, edited and approved the final text.
